# Influence of Vitamins on Secondary Reactive Oxygen Species Production in Sera of Patients with Resectable NSCLC

**DOI:** 10.3390/diseases4030025

**Published:** 2016-07-14

**Authors:** Thierry Patrice, Bertrand Rozec, Alexis Sidoroff, Yvonnick Blanloeil, Philippe Despins, Christian Perrigaud

**Affiliations:** 1 Anesthesiology and Intensive Care, Laënnec Hospital, 44093 Nantes, France; bertrand.rozec@chu-nantes.fr (B.R.); yvonnick.blanloeil@chu-nantes.fr (Y.B.); 2 Department of Dermatology and Venereology, Medical University of Innsbruck, Innsbruck, A-6020, Austria; toupai.sasuke@gmail.com; 3 Thoracic Surgery, Laënnec Hospital, 44093 Nantes, France; philippe.despins@chu-nantes.fr (P.D.); christian.perrigaud@chu-nantes.fr (C.P.)

**Keywords:** NSCLC, singlet oxygen, reactive oxygen species (ROS), vitamin, glutathione

## Abstract

Background: Singlet oxygen (^1^O_2_) oxidizes targets through the production of secondary reactive oxygen species (SOS). Cancers induce oxidative stress changing with progression, the resulting antioxidant status differing from one patient to the other. The aim of this study was to determine the oxidative status of patients with resectable Non-Small cell lung cancers (NSCLC) and the potential influence of antioxidants, compared to sera from healthy donors. Materials and Methods: Serum samples from 10 women and 28 men, 19 adenocarcinomas (ADK), 15 patients N1 or M1 were submitted to a photoreaction producing ^1^O_2_. Then, samples were supplemented with vitamins (Vit C, Vit E), or glutathione (GSH). Results: Squamous cell carcinomas (SCC) and metastatic SCCs induced a lower SOS rate. While Vit C increased SOS in controls as in patients with metastases, Vit E or the combination of Vit E and C strongly reduced SOS. GSH alone lightly decreased SOS in controls but had no effect in patients either alone or combined with Vit C. Conclusion: In “early” lung cancers, SOS are comparable or lower than for healthy persons. The role of Vitamins varies with gender, cancer type, and metastases. This suggests that an eventual supplementation should be performed on a per-patient basis to evidence any effect.

## 1. Introduction

A number of diseases are oxidative stress-related, including cancers that are prone to inducing oxidative stress at any step of their development from induction to metastasis [1,2], this oxidative stress even being increased in the particular case of cancer-induced pulmonary obstruction [3]. In addition, most anticancer treatment procedures act through oxidative processes and are associated with significant mortality and morbidity or an inflammatory response. Among the factors that influence this inflammatory response, oxidative stress has been shown to play a major role, being related to either the disease or the patient. This oxidative stress results in the formation of reactive oxygen species (ROS) and peroxides originating from various cellular and enzymatic sources and leads to the depletion of plasma physiological antioxidants, increasing peroxidation and causing additional tissue damage [4]. In addition to ROS, singlet oxygen (^1^O_2_)—a molecular excited form of oxygen that is generated during inflammatory reactions or during photoreactions [5,6]—can undergo de-activation to produce secondary ROS as hydroxyls, alkoxyls, peroxyls, superoxide anion, and peroxides (SOS), and is strongly oxidant for many biological targets, either directly or through the formation of SOS [7,8]. We demonstrated that resistance to SOS was decreased in advanced cancers—experimentally and in patients [9,10]—and recently, a correlation between antioxidant, Vitamins (Vit) and inflammation biomarkers had been observed in patients with metabolic syndrome [11], something that could to some extent influence cancer growth. Whereas several studies aimed at reinforcing antioxidant defense during diseases or deficiencies, using vitamins has shown a positive effect. Others have reported a lack of benefit using vitamin C (Vit C, [12]), vitamin E (Vit E, [13,14]), and combinations of these agents [15], including in large prospective studies [16,17]. In the present paper, we studied the influence of substances classically prescribed for their anti-oxidative properties on SOS production and de-activation in sera from 28 men and 10 women with Non-Small Cell Lung Cancers (NSCLC), consecutively recruited. These cancers were all considered as being resectable, although one node or metastasis had been detected in 15 patients. Primary and secondary lesions had been resected. Sera obtained pre-operatively underwent a photooxidation to convert the normally present O_2_ into singlet oxygen (^1^O_2_) as a source of ROS. Vitamin C, Vit E, reduced glutathione (GSH), and combinations of Vit C with GSH (as GSH reduces oxidized Vit C [18,19]) and with Vit E (as a combination of Vit C and E is thought to be more efficient [20]) were added to sampled sera immediately after ^1^O_2_ photo production in order to examine potential different SOS productions according to patients.

The aim of the study was to evidence different resistance to SOS in resectable NSCLC according to pathology, the presence of metastases, and after the addition of Vit C, Vit E, reduced-glutathione, or combinations of these.

## 2. Materials and Methods

The study was conducted according to the protocol (NTS 2006-02) for the collection of healthy sera and was approved by the Nantes University Hospital ethics committee (HD-07/1519) in accordance with the Helsinki declaration (1964/2000). All donors were duly informed of the aim of blood sampling.

### 2.1. Patients and Controls

Thirty-eight patients eligible for surgery (10 women, mean age 60.7, range 47–75; and 28 men, mean age 61, range 46–74) were consecutively recruited who had been diagnosed with T1 or T2 NSCLC (19 adenocarcinomas (ADK), 19 squamous cell carcinomas (SCC)) between December 2008 and December 2010 (Table 1), and the forced expiratory volume in one second (FEV 1) measured as a reduced paO2 could modify patients’ resistance to oxidative stress. Fifteen had been diagnosed with one node or one metastasis (Metas) either preoperatively or during the surgical procedure. All blood samples had been obtained the day before surgery. None of patients admitted to taking any antioxidant, having smoked or drank alcohol during the 2 h before blood collection, which occurred between 8 and 11 am for all patients and donors, before any food intake. All these requirements are valid for any routine blood donor of the institution. Follow-up had been retrospectively analyzed 12 and 24 months after surgery. For antioxidant assays, samples had been pooled according to gender or the presence of node and metastasis. Blood samples sent to our lab were identified only by numbers, so that sample processing had been performed without knowing the patients’ characteristics. The study could thus be considered as semi-blinded.

Volunteer adult routine blood donors—25 women (mean age 40) and 25 men (mean age 39, *p* < 0.001 as compared to patients)—were enrolled consecutively, randomly, and blindly (thus excluding the possibility of any clinical examination for all—i.e., body mass index determination or date of menstruation for women but limiting bias linked to food regimens). Blood sampling was carried out in the same conditions as for the patients and sera pooled according to gender. None of the healthy donors had ever been previously diagnosed with a severe disease. Controls consisted of a longitudinal pool of sera that had been used for 5 years (100% or 1 (22 women, mean age 40.2 years)) and used as an internal reference to avoid any device fluctuation. For this reason, the ratio of healthy donors of the present study is 0.85 +/− 10% as improvements in the technology had been made.

### 2.2. Blood Sampling

Ten milliliters of blood were drawn from the left radial artery line into sterile clot act dry tubes (Venosafe VF-054SP, Terumo Europe, Leuven, Belgium). Haemolysis during blood sampling and hemoglobin aspiration during serum collection under sterile conditions were avoided as much as possible. Sera samples were frozen at −20 °C until processed. The period between blood sampling and freezing did not exceed 40 min. On the day of experiments, sera were thawed to 0 °C, pooled, and aliquoted in 0.5 mL tubes (micro centrifuge tubes VWR, 94126 Fontenay-sous-Bois, France).

### 2.3. Assay for SOS Production Analysis

The principle of the measurement [21] is to produce a standardized amount of photodynamically-induced ^1^O_2_ using Rose Bengal (RB, (CAS N° 632-69-9), 5 µg/mL final concentration) as a source of ^1^O_2_ under light exposure (514 nm, 500 mW, 20 J/cm^2^) in the serum to be assayed (25 µL in 450 µL water for injections) and to measure the speed of neutralization of secondary ROS and/or peroxides. Light was delivered from an argon ion laser by means of an optic fibre (400 µm core diameter). The speed of neutralization of secondary ROS and/or peroxides was then analyzed by the DCFH/DCF system, with activated DCFH added to each assayed sample immediately after the end of light delivery and in a standardized manner. The area under the curve (AUC) for the production of fluorescent DCF upon exposure to ROS (excitation 488 nm, emission 525 nm) was measured over time by means of pixel number calculation via image analysis software throughout a standardized computer procedure for each blood sample and a calculated ratio patient/control. Absorption spectra were obtained using a Techcomp 8500 absorption spectrophotometer (Techcomp, Hong Kong, China) after dilution in water for injections (5% serum). The level of hemolysis was evaluated from the 413-nm peak absorption and the baseline by the 650-nm absorption, where there is normally a minimal absorption. Only sera with absorption less than 0.3 at 413 nm were considered.

Vit C (CAS N° 50-81-7), Vit E (alpha-tocopherol, CAS N° 10191-41-0), GSH (CAS N° 70-18-8) were obtained from Sigma Aldrich (38070 Saint-Quentin-Fallavier, France). Vit C and GSH were dissolved in sterile water. Vit E was dissolved in absolute ethanol (0.5 mL) and then in a mixture of Tween^®^ 20 (0.1 mL Water was used for all successive dilutions. In the present assay, final concentrations of 12, 6, and 3 µg/mL to keep final absorbance lower than 0.3 optical density were added (25 µL) to irradiated solutions containing sera and RB, immediately after the end of light delivery, incubated for 5 s, before DCFH addition and subsequent fluorescence determination. When using Vit E, the influence of solvents was subtracted from the effects of antioxidants plus solvents. 

Quantitative determination of total 25-OH Vit D levels were measured using commercially available competitive electrochemiluminescence protein binding assay, onto Cobas 6000 Analyzer (Roche Diagnostic, Meylan, France). The assay employs a Vit D binding protein (VDBP) as capture protein, which binds to both 25-OH D3 and 25-OH D2 (80% analytical specificity to D2 and 98% analytical specificity to D3). After patient sample (15 µL) incubation with pre-treatment reagent—which releases bound 25-OH Vit D from the VDBP sample—is incubated with ruthenium-labelled VDBP, creating a complex between the 25-OH Vit D and the ruthenylated VDBP. Addition of streptavidin-coated microparticles and 25-OH Vit D labelled with biotin forms the ruthenium-labelled Vit D binding protein and the biotinylated 25-OH Vit D. The assay is standardized using NIST SRM 972 for controls and NIST SRM 2972 for calibration. Between-day precision was CV = 4.4% and 5.2% at mean concentrations of 21 and 34 ng/mL, respectively, using quality control material provided by Roche Diagnostics. Using this assay, the domain of measure is 3–70 ng/mL (7–175 nmol/L).

Each patient measurement included two controls: a man or woman sample as well as a man plus woman control (longitudinal control allowing a comparison between studies and controls). Each measurement had been performed as a quadruplicate and performed twice, giving eight values. Sera from each pool were treated as for patients, with either each respective solvent at the same concentration and a replicate sample receiving 25 µL of water. The direct effects of solvents or solvents plus chemicals on DCF (produced by UV irradiation of DCFH for 60 min) absorbance and fluorescence was also measured. Patients were asked for past and actual tobacco consumption, assayed for forced expiratory volume in 1 s (FEV1), endogenous Vit or GSH serum concentration, viral status (HIV and Hepatitis B), HDL-C, LDL-C, total cholesterol, triglycerides, HbA1c, Na, K, Ca, total proteins and albumin, glucose, urea, creatinine, uric acid, total and conjugated bilirubin, and C-reactive protein in order to verify the absence of abnormal situations. 25(OH)D Vit had also been measured, as it represents the body Vit D reserves. Blood counts, including platelets, Hb, hematocrit, Mean Corpuscular Volume, Mean Corpuscular Hemoglobin Concentration, Mean Corpuscular Hemoglobin, as well as blood typing, were also performed to detect an anemia likely to impair results.

### 2.4. Statistical Analysis

The Mann–Whitney U test was used for data comparisons, the Chi-square test for group comparisons. *P* values *p* < 0.05 were considered statistically significant. Five to eight measurements were performed per data point.

## 3. Results

Profiles of DCF fluorescence evolution as well as the AUC values were similar to previous data [21]. From preliminary data, we noted that the evolution of DCF fluorescence after photosensitization of one given human serum vial by RB under similar conditions was found to be highly reproducible [21]. In the present study, the standard deviation (SD) was always less than 0.05 and rarely above 0.03 for a given compound added to a given serum. There was no statistical difference between male or female controls, although there was a trend towards a lower SOS production in male sera (*p* < 0.08). A statistically higher standard error in cancer patients (0.45) as compared to normal samples (0.23) was noted (*p* < 0.05). The mean SOS production was similar to controls. However, SOS were lower for SCC than ADK and lower in each group when metastases had been diagnosed (Table 1).

However, metastases decreased SOS in SCC-bearing men or women, but SOS was significantly more decreased in ADK-bearing men as compared to women. A lower SOS production (Table 2) was associated with a poor SCC prognosis (*p* < 0.05) and as compared to ADK (*p* < 0.04) and a low FEV 1, underlining a different evolutive pathway for each type of tumor.

**Figure 1:**
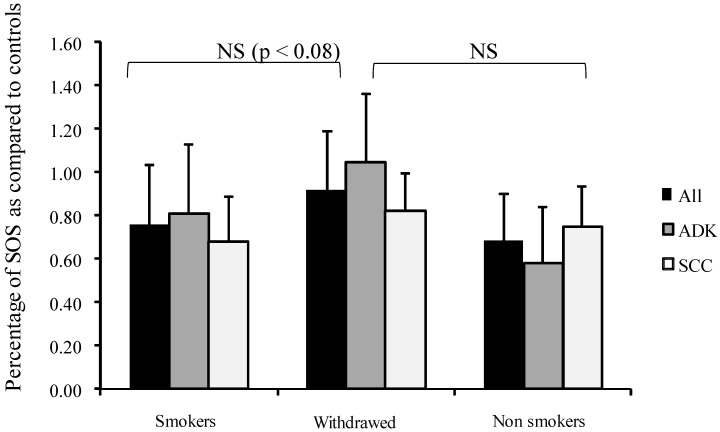
Influence of smoking on percentage of secondary oxygen species (SOS) after a photoinduced calibrated singlet oxygen delivery in T1/T2 NSCLC as compared to healthy controls. SOS produced were calculated from the area under the curve of DCF fluorescence resulting from the DCFH oxidation induced by a Rose Bengal-mediated photoreaction (Rose Bengal 5 µg/mL, 514 nm, 20 J/cm^2^) in sera (5% in water). Presumed healthy sera were pooled from a series of 23 male and 23 female samples. For all sera, reference was from a longitudinal pool of 53 men and women samples. Percent was measured from a 66 min analysis after DCFH addition by comparison with untreated sera of each group. Dark bars: whole group, Medium grey: patients with adenocarcinomas, Light grey: patients with squamous cell carcinomas. NS: Non-significant.

Interestingly, SOS appeared higher (but not significantly) in tobacco-withdrawn patients than in active smokers (Figure 1).

**Figure 2:**
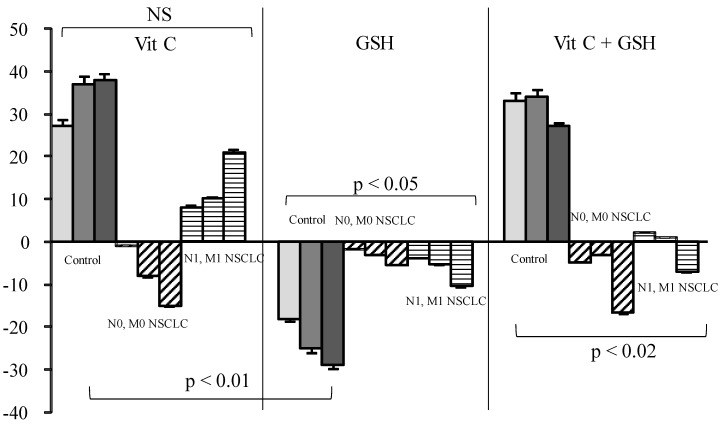
Influence of Vitamin (Vit) C, reduced glutathione (GSH), or a combination of the two on percentage of secondary oxygen species (SOS) after a photoinduced calibrated singlet oxygen delivery in T1/T2 non-small cell lung cancers as compared to healthy controls. For all sera, reference was from a longitudinal pool of 53 Men and women samples. Anti-oxidative compounds (3 (left bars), 6 (middle), 12 (right) µg/mL) were added immediately after the end of light delivery and gently rocked for 5 s before DCFH addition and fluorescence recording. NS: Non significant.

Vit C or Vit C plus GSH increased SOS production in healthy sera from 29% to 33% for doses ranging from 3 to 12 µg/mL when GSH decreased SOS (Figure 2).

**Figure 3:**
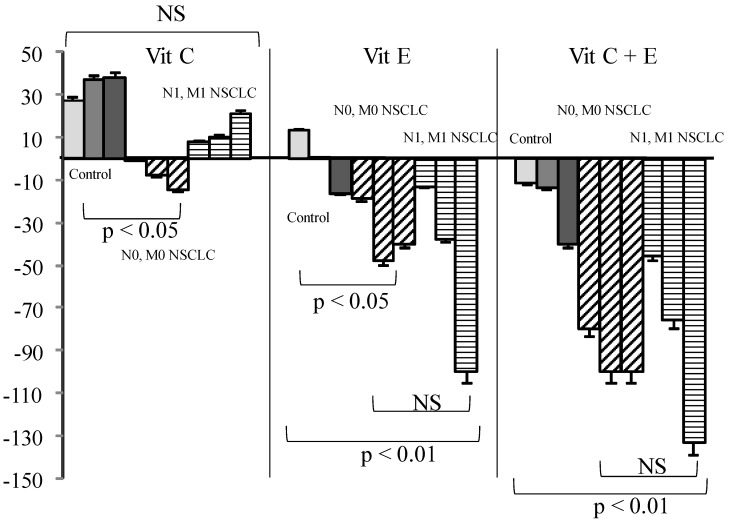
Influence of Vit (Vit) C, Vit E, or a combination of the two on percentage of secondary oxygen species (SOS) after a photoinduced calibrated singlet oxygen delivery in T1/T2 non-small cell lung cancers as compared to healthy controls. Percent were measured from a 66 min analysis after DCFH addition by comparison with untreated sera of each group. For all sera, reference was from a longitudinal pool of 53 Men and women samples. Anti-oxidative compounds (3, 6, 12 µg/mL) were added immediately after the end of light delivery and gently rocked for 15 s before DCFH addition and fluorescence recording. NS: non-significant.

In cancer patients either with or without metastases, Vit C or GSH addition had nearly no effect. On the contrary, Vit E alone or associated with Vit C significantly reduced SOS production as compared to what was observed in healthy controls (Figure 3, *p* < 0.01).

Vit D (Table 3) was found decreased in cancer patients as compared to controls, particularly in SCC and SCC with metastases (0.05 < *p* < 0.001). However, no correlation could be found between Vit D status and the ratio of SOS production.

Chemicals or solvents added without sera did not induce any change in DCF fluorescence or absorbance (in the absence of RB or light), suggesting that the observed effects were not linked to a direct effect on DCF, light scatter, or impaired transmission during illumination. When ethanol was used as a solvent for Vit E, a mild dose-dependent increase in SOS production was observed. The AUC calculated from the fluorescence obtained by adding DCF to sera containing Vit E or Vit E and C were re-calculated taking into account Vit E solvent alone used for each respective Vit E concentration. All biochemical controls were, according to our inclusion criteria, within the normal range of values of our institution for women and men including baseline Vit concentrations, glucose, proteins, and lipids. Estrogen was 35 pg/mL in the female pool and 19 in males.

## 4. Discussion

Oxidative stress, namely the imbalance between ROS production and deactivation, can result in auto-oxidation of glucose, energy production disorders, shifts in redox balances, and reduced or impaired activities of enzymes participating in antioxidant defense [22,23]. An overproduction of ROS or SOS may thus induce deleterious and unpredictable complications [4]. The direct measurement of ROS is almost impossible on a routine basis or in vivo in patients. Furthermore, the measurement of all antioxidant molecules separately is difficult, if not impossible. Global antioxidant measurements may be more physiologically relevant because they take account of potential synergistic or antagonistic [24] effects of antioxidants that can be very different in nature: vitamins, albumin, enzymes, more specific molecules such as glutathione, but also non-specific compounds with antioxidant properties such as uric acid or bilirubin. In addition, oxidative stress following a metabolic impairment does not evolve linearly as during cancer growth [10] and therefore antioxidants should not be given blindly whatever the time of administration. What is said to be an antioxidant in a healthy person could be a strong pro-oxidant in other circumstances and induce deleterious effects during certain periods of time.

In previous studies, we measured different amounts of SOS, after ^1^O_2_ production in serum, between one healthy individual to another within a given species, according to hemolysis or age [21], during various diseases known to modify oxidative stress, as well as in patients with cancers [7]. Cancers are accompanied with various and unpredictable metabolic disorders; i.e., through Warburg effect-related pathways [25]. An over production of SOS in patient sera during cancers had been correlated with a poor prognosis [26]. In a previously published series of patients, three had an advanced lung cancer with a mean SOS production of 1.34, thus much over the upper limit of our normal range [7]. Observed differences between early and advanced cancers fitted to experimental data obtained in mice [8] showed that soon after graft, squamous cell cancers induced an SOS decrease after ^1^O_2_ delivery, when it rose just before entrance into cachexia until death. A gap between the mean ages of healthy donors control and NSCLC-bearing patients (40 versus 60) was noted. However, this seems to us to be of little influence, since aging between 20 and 60 induces a decrease in SOS production [21]. Therefore, measuring a decrease in SOS production in sera from patients with resectable NSCLC induces a limited per default error (0.1) as compared to the mean age of our healthy cohort. Another reason is that it is extremely rare to find really healthy persons after 60 years of age, the impact of their various diseases on oxidative stress being unpredictable.

The present study was thus aimed at verifying whether SOS production after ^1^O_2_ delivery in the sera of resectable NSCLC-bearing patients differed according to the initial pathology and as compared to SOS produced in sera from healthy patients. We also verified whether the relative anti-oxidative effect of C, E, GSH or their combinations—known to biochemically cooperate against oxidative stress—might be efficient on sera from patients bearing “early” cancers in comparison to sera from healthy donors. From this point of view, it was not the aim of this paper to study the influence of well-described and widely used compounds, but rather to verify that we were able to find effects described using our method. Main figures of our results showed that SOS production was normal or decreased in all groups of patients but that SOS were more decreased in sera of patients with “early” SCC, thus confirming the results observed in mice [8]. The presence of metastases influenced SOS inversely in men and women with adenocarcinomas: SOS was normal in women bearing adenocarcinomas and lowest in men and women having metastatic SCC.

SOS production did not correlate with Vit D content, which was decreased in all groups, but the highest mean Vit D concentration was found in patients with metastatic adenocarcinomas (N = 8) and the lowest one in patients with metastatic squamous cell carcinomas (N = 7)—patients also having the lowest rate of SOS. The role of Vit D deficiency in cancers had been suspected for a long time [27], although not firmly established [28] for clearly limited types of patients [29], and possible mechanistic pathways are numerous [30]. Heterogeneous Vit D deficiencies could, however, be related to its hyper-oxidation in the presence of ROS, as Vit D may act as a weak antioxidant or ^1^O_2_ scavenger, when ROS or SOS vary according to overall patients’ defense, cancer types, or even FEV1.

In contrast to what was expected, Vit C induced a SOS increase in control samples and in metastatic patients, although minor, but was nearly ineffective in all other groups of patients. GSH alone markedly decreased SOS in controls, but this effect was annihilated in cancer patients either when used alone or in combination with Vit C. On the contrary, Vit E induced only a mild reduction in healthy sera but a strong SOS decrease in cancer patients, either alone or in combination. Vit C also named ascorbate or ascorbic acid has never been proven useful in any blinded supplementation assay, including when associated with other vitamins [12,16,31]. From our results, and supported by other findings [13,32,33], Vit C is not considered to act as an in vivo antioxidant—at least in healthy conditions—but rather acts as a co-factor for other [34] antioxidants such as GSH (which is intracellular [35,36,37]) or Vit E [13,15]. This is coherent with literature showing that a reducing agent such as Vit C may produce superoxide radicals and eventually hydroxyl radicals [38] under aerobic conditions. This role of Vit C has previously been reported in studies on the impact of the radiomimetic bleomycin on cell models [39] and administration of Vit C, with other GSH failed to ameliorate free radical-mediated damage. The anti-oxidative effect of GSH, which is both a “regenerator” of Vit C and a potent antioxidant, was almost completely annihilated by Vit C. This pro-oxidant effect of Vit C could explain the lack of efficacy of the administration of high doses [33].

Vitamin E, although poorly efficient in healthy sera, became a powerful antioxidant when used in combination with Vit C when administration of Vit E has been shown to be protective in a number of studies [15] involving patients. Vit C has been demonstrated to recycle Vit E [15] from its alpha-tocopheroxyl radical in the case of excessive oxidative stress. This would explain the stronger efficacy of the combination in cancer patients, as our results demonstrate that the anti-oxidative efficacy of Vitamins varies widely according to gender between healthy and cancer sera. This had been also noted from the Caret study for Vit A [17]. This could be related to an overconsumption of antioxidants present physiologically in healthy persons during pathological situations—i.e., tumor growth or submission to a risk factor like tobacco. The non-significant SOS increase in withdrawn smokers had been also found in other non-cancer-related studies [40]. One could imagine that smoking induces mobilization of non-spontaneously present antioxidative defense to maintain homeostasis, stopping with smoking arrest but induced metabolic damages persisting. The effects of vitamins C and E are greater in patients with metastases, thus presumed to have a larger “overall” tumor volume. It is possible, however, that a decreased anti-oxidative capacity increases the risk for a tumor to give metastases. If it were the case, studies involving an anti-oxidant supplementation should at first determine the exact needs of a patient. As a higher SOS production had been associated with a worse prognosis in all our studies, regardless of the patients analyzed, a better efficacy of an antioxidant supplementation would suggest a better compensation of cancer-induced deleterious effects. One could consider that under cancer developments, antioxidants are first balanced by normal defense that are then over-consumed. At this step, a supplementation helps recover a “normal” oxidative status, eventually protecting from additional SOS-induced adverse effects. In any case, a blind supplementation could lead to deleterious effects—i.e., stimulating tumor growth if not fitting with the real needs of the patient. However, a strong variation had been found for both SOS production and efficacy of Vit addition to sera. Not only individuals, but also pathology may influence this efficacy, since SOS is significantly decreased in patients bearing SCC as compared to adenocarcinomas. This is another argument in favor of a precise quantitative appreciation of antioxidant needs before any supplementation. 

The lack of specificity of the DCFH–DCF system [41] that we routinely use enables the detection not only of primary radicals and radical ions but also the whole family of oxi-radicals and peroxides, in real-time [42]. What makes our method particularly sensitive in the detection of SOS is that it bypasses the spin interdiction that makes oxygen unable to directly oxidize biological molecules such as glucose or lipids. When oxygen is excited into its singlet state, it can directly oxidize targets, and then the deactivation will follow the physiological routes of deactivation [7]—something impossible for tests requiring the addition of a chemical likely to react with a given type of compound (such as lipids) to produce ROS.

The definition of an anti-oxidative activity for a chemical is usually based on in vitro modifications of the redox potential of solutions, measurements often made in the absence of serum, given that proteins are strongly anti-oxidative [43] and may therefore mask the anti-oxidative properties. In addition, antioxidants acting in vitro may induce difficult to detect unpredictable or adverse indirect effects in the animal models used. Similarly, measuring anti-oxidative activity in conditions similar to in vivo may clarify the potential utility of providing supplements to patients, particularly during situations of severe oxidative stress—i.e., cancer, aging, or diabetes mellitus [44,45], during which defense against ROS is markedly impaired.

The potential of improving patient care by decreasing or even preventing oxidative stress during diseases such as cancers remains an important goal. Recently, the potential utility of providing Vit C and E [44] had been underlined [13]. Our results suggest that Vit C supplementation should be prescribed carefully and adapted to the Vit E concentration in order to avoid an increased SOS production. Our study emphasizes the importance of continued research aimed at neutralizing oxidative stress. As different results could be noted in patients, future research should thus focus on adapted antioxidant protocols on a per-patient basis. This would require a fast and reliable test to measure anti-oxidative defense that could also provide information about the kind of mechanism involved in the deficit of a given antioxidant. This point is of importance, since one could imagine that a similar value in two persons may correspond to different anti-oxidative situations that could be compensated differently in each, to a certain extent. Consequently, the beneficial effects of Vitamins—most often used as a single-compound supplement—remain controversial, as does their long-term use as supplements [31,45]. Such studies blindly recruiting persons producing high ROS as well as others producing less ROS could have a reduced statistical sensitivity. In the future developments of our method, photo-oxidized sera could be added to pre-positioned wells containing various relevant anti-oxidants as single compounds or as mixtures, and calculation of the AUC could directly indicate the most relevant compound and its concentration.

## Figures and Tables

**Table 1:** Influence of pathology or metastases on the percentage of secondary reactive oxygen species after a photoinduced calibrated singlet oxygen delivery in T1/T2 resectable NSCLC as compared to healthy controls. N: Number of patients, Ratio as compared to healthy controls (Standard error in brackets). Reference is calculated from a longitudinal pool of men and women’s sera samples (Ratio of 1). ADK: Adenocarcioma, SCC: Squamous cell carcinoma, N: Node, M, Metastase.

	N	Whole	N	Women	N	Men
**Age**		61.3		60.7		61
**Whole cohort**	38	0.84 (0.45)	10	0.79 (0.36)	28	0.82 (0.46)
**Whole N0Mo**	23	0.83 (0.2)	4	0.63 (0.2)	19	0.87 (0.2)
**Whole N1 or M1**	15	0.76 (0.2)	5	0.9 (0.3)	10	0.68 (0.4)
**ADK**	19	0.9 (0.5)	8	0.83 (0.4)	11	0.88 (0.6)
**ADK N0M0**	11	0.87 (0.6)	4	0.6 (0.47)	7	1.07 (0.7)
**ADK N1 or M1**	8	0.84 (0.1)	4	1.04 (0.3)	4	0.65 (0.17)
**SCC**	19	0.77 (0.35)	2	0.63 (0.17)	17	0.78 (0.4)
**SCC N0M0**	12	0.78 (0.4)	0	-	12	0.72 (0.2)
**SCC N1 or M1**	7	0.69 (0.17)	2	0.62	5	
**Controls**	50	0.85 (0.23)		0.83 (0.21)		0.77 (0.24)

**Table 2:** Influence of evolution or of forced expiratory volume in one second on percentage of secondary reactive oxygen species after a photoinduced calibrated singlet oxygen delivery in T1/T2 resectable non-small cell lung cancers as compared to healthy controls. N or n: Number of patients, Ratio as compared to healthy controls (SE in brackets). Reference is calculated from a longitudinal pool of men plus women sera samples (Ratio of 1). ADK: Adenocarcioma, SCC: Squamous cell carcinoma, N/n: Number of patients in the whole cohort or dead or evolutive subgroup (n), CR: complete response, FEV 1, Forced expiratory volume in one second.

	N	n	Dead or Evolutive	CR	<80	FEV 1 80 < M < 100	100
**Whole**	38	10	0.85 (0.6)	0.7 (0.35)	0.87 (0.57)	0.8 (0.34)	0.8 (0.3)
**ADK**	19	5	1.02 (0.9)	0.83 (0.36)	1.46 (0.9)	0.82 (0.4)	0.64 (0.14)
**SCC**	19	4	0.65 (0.15)	0.87 (0.4)	0.75 (0.4)	0.64 (0.06)	0.90 (0.3)

**Table 3:** Correlation between Vitamin D (ng/mL) and percentage of secondary reactive oxygen species after a photoinduced calibrated singlet oxygen delivery in T1/T2 resectable non-small cell lung cancers as compared to healthy controls. N: Number of patients, Ratio as compared to healthy controls (SE in brackets). Reference is calculated from a longitudinal (Historically the same for 5 years) sample. K: NSCLC studied, W: women, M: men, ADK: Adenocarcioma, SCC: Squamous cell carcinoma, Meta +/−: Presence or absence of Metastases.

	Vitamin D	Ratio (Control)
**Control**	32.2	0.8 (1)
**Women**	25.5 (0.05)	0.83
**Men**	26.9 (0.04)	0.77
**All K**	14.12 (10)	0.84
**All K W**	13 (9.9)	0.79
**All K M**	14.51 (11)	0.82
**All meta −**	11.75 (6.5)	0.83
**All meta +**	20.26 (15)	0.76
**ADK**	16.24 (13)	0.9
**ADK meta −**	11.42 (8.3)	0.86
**ADK meta +**	22.51 (15)	0.84
**SCC**	11.24 (5.3)	0.77
**SCC meta −**	12.76 (5.1)	0.78
**SCC meta +**	7.2 (5.3)	0.69

## Abbreviations

The following abbreviations are used in this manuscript:

^1^O_2_	Singlet oxygen
ADK	Adenocarcinoma
AUC	Area Under the Curve
DCF	Dichlorofluorescein (oxidized, fluorescent)
DCF-DA	Dichlorofluorescein Diacetate
DCFH	Dichlorofluorescein (reduced, non fluorescent)
FEV 1	Forced expiratory volume in one second
GSH	Reduced glutathione
Met	Metastase
NSCLC	Non-small cell lung cancer
RB	Rose Bengal
ROS	Reactive Oxygen Species and peroxides
SCC	Squamous cell carcinoma
SD	Standard Deviation
SOS	Secondary Reactive Oxygen Species and peroxides
Vit C	L-ascorbic acid, Vitamin C
Vit D	Vitamine D2 (ergocalciferol) and D3 (cholecalciferol) (25-OH)
Vit E	Alpha tocopherol, Vitamin E
